# Antrocin Sensitizes Prostate Cancer Cells to Radiotherapy through Inhibiting PI3K/AKT and MAPK Signaling Pathways

**DOI:** 10.3390/cancers11010034

**Published:** 2018-12-31

**Authors:** Yu-An Chen, David T. W. Tzeng, Yi-Ping Huang, Chun-Jung Lin, U-Ging Lo, Chia-Lin Wu, Ho Lin, Jer-Tsong Hsieh, Chih-Hsin Tang, Chih-Ho Lai

**Affiliations:** 1 Graduate Institute of Basic Medical Science, School of Medicine, China Medical University, Taichung 40402, Taiwan; yachen.cmu@gmail.com (Y.-A.C.); yphuang@mail.cmu.edu.tw (Y.-P.H.); 2 Department of Urology, University of Texas Southwestern Medical Center, Dallas, TX 75390, USA; Chun-Jung.Lin@UTSouthwestern.edu (C.-J.L.); U-Ging.Lo@utsouthwestern.edu (U.-G.L.); jt.hsieh@utsouthwestern.edu (J.-T.H.); 3 Department of Microbiology and Immunology, Department of Biochemistry, College of Medicine, Chang Gung University, Taoyuan 33302, Taiwan; clwu@mail.cgu.edu.tw; 4 School of Life Sciences, The Chinese University of Hong Kong, Hong Kong 999077, China; allqwdd@gmail.com; 5 Department of Neurology, Chang Gung Memorial Hospital, Linkou 33305, Taiwan; 6 Department of Life Sciences, National Chung Hsing University, Taichung 40227, Taiwan; hlin@dragon.nchu.edu.tw; 7 Graduate Institute of Biomedical Sciences, China Medical University, Taichung 40202, Taiwan; 8 Department of Nursing, Asia University, Taichung 41354, Taiwan; 9 Molecular Infectious Disease Research Center, Department of Pediatrics, Chang Gung Memorial Hospital, Linkou 33305, Taiwan

**Keywords:** prostate cancer, radiotherapy, radioresistance, antrocin

## Abstract

Radiotherapy is one of the most common treatment options for local or regional advanced prostate cancer (PCa). Importantly, PCa is prone to radioresistance and often develops into malignancies after long-term radiotherapy. Antrocin, a sesquiterpene lactone isolated from *Antrodia cinnamomea*, possesses pharmacological efficacy against various cancer types; however, its therapeutic potential requires comprehensive exploration, particularly in radioresistant PCa cells. In this study, we emphasized the effects of antrocin on radioresistant PCa cells and addressed the molecular mechanism underlying the radiosensitization induced by antrocin. Our results showed that a combination treatment with antrocin and ionizing radiation (IR) synergistically inhibited cell proliferation and induced apoptosis in radioresistant PCa cells. We further demonstrated that antrocin downregulated PI3K/AKT and MAPK signaling pathways as well as suppressed type 1 insulin-like growth factor 1 receptor (IGF-1R)-mediated induction of β-catenin to regulate cell cycle and apoptosis. Using xenograft mouse models, we showed that antrocin effectively enhanced radiotherapy in PCa. Our study demonstrates that antrocin sensitizes PCa to radiation through constitutive suppression of IGF-1R downstream signaling, revealing that it can be developed as a potent therapeutic agent to overcome radioresistant PCa.

## 1. Introduction

Prostate cancer (PCa) is one of the most common male cancers in the Western world, and its incidence is currently rising in Asia. Several modalities are generally employed in PCa treatment including surgery, radiotherapy, hormone therapy, chemotherapy, and bone-directed treatment [1]. Radiotherapy is a non-invasive, low-risk option for PCa treatment [2], however, PCa often becomes resistant to radiation after long-term radiotherapy and can develop into malignancies that fail to be treated with radiotherapy [3]. Therefore, discovering alternative therapies for enhancing radiosensitivity in PCa is urgently required.

Differentially expressed in ovarian carcinoma 2/disabled-2 (DOC-2/DAB2 or DAB2IP) is also known as ASK1-interacting protein-1, a novel member of the Ras GTPase-activating protein family [4]. DAB2IP is a member of the disable gene family and exhibits tumor inhibitory activity [5]. Increased DAB2IP expression inhibits the growth of several cancer cells, suggesting that it functions as a tumor suppressor [6,7]. Loss of DAB2IP expression promotes PCa development [8,9] and confers resistance to radiation-induced apoptosis, thus inducing a cancer stemness phenotype [10,11].

Antrocin, a sesquiterpene lactone, was isolated from *Antrodia cinnamomea* and has been used as a dietary supplement for cancer prevention and hepatoprotection in Asia [12]. The chemistry of *A. cinnamomea* has been extensively studied, leading to the identification of more than 100 secondary metabolites [12]. *A. cinnamomea* has been used in traditional Chinese medicine for the treatment of drug intoxication, abdominal pain, food poisoning, and cancer [13]. Recently, antrocin has been reputed as the most potent component that contributes to the pharmacological efficacy of *A. cinnamomea* [12]. In addition, antrocin has exhibited a potent antagonist effect against a variety of cancers including breast, lung, liver, and colon cancers [14]. Moreover, antrocin has been demonstrated to inhibit the ERK/c-Fos/MMP-2, Akt/mTOR/GSK-3β/NF-κB, and JAK/STAT3 signaling pathways, contributing to its suppressive effect against several cancers [14,15,16]. However, whether antrocin can enhance radiosensitivity in PCa cells still requires further investigation. In this study, we explored the effect of antrocin on radioresistant PCa cells and identified its mechanism of action on different signaling pathways. Furthermore, we also assessed the effects of antrocin treatment in xenograft tumor models. The results demonstrate that antrocin could be developed as a potent therapeutic agent to overcome radioresistant PCa.

## 2. Materials and Methods

### 2.1. Antibodies and Reagents

Antibodies against PARP, BAX, cleaved-caspase 3, cleaved-caspase 9, CDK2, phospho-CDK2, p16, p21, and cyclin D1 were purchased from Proteintech (Chicago, IL, USA). Antibodies specific to IGF-1R, phospho-IGF-1R, JNK, phospho-JNK, p38, phospho-p38, ERK1/2, phospho-ERK1/2, PI3K p85, PI3K p110, AKT, phospho-AKT, GSK3-β, phospho-GSK3-β (Ser9), β-catenin, and phospho-β-catenin were purchased from Cell Signaling (Danvers, MA, USA). Antibodies against Bcl-2, β-actin, and rabbit horseradish peroxidase (HRP)-conjugated antibody were purchased from Santa Cruz Biotechnology (Santa Cruz, CA, USA). All other reagents were purchased from Sigma-Aldrich (St. Louis, MO, USA).

### 2.2. Cell Culture

The shRNA system (pGIPZ-lentiviral-shRNAmir from Open Biosystems, Huntsville, AL, USA) was used to knockdown (KD) endogenous DAB2IP in various prostate epithelial cell lines: knockdown (shDAB2IP) cells were selected under puromycin and G418 [9]. All lines of prostate cells were kindly provided by Dr. Jer-Tsong Hsieh (Department of Urology, University of Texas Southwestern Medical Center, Dallas, TX, USA). PC-3, DU145, and LNCaP cells were maintained in RPMI1640 supplemented with 5% fetal bovine serum (FBS) (Hyclone, Logan, UT, USA) in a humidified atmosphere containing 5% CO_2_. LAPC4 cells were cultured in Dulbecco’s Modified Eagle Medium (DMEM) (Gibco, Grand Island, NY, USA) supplemented with 5% FBS. PZ-HPV-7, an immortalized cell line derived from the peripheral zone of a benign prostate, was maintained in RPMI1640 supplemented with 10% FBS.

### 2.3. Preparation of Antrocin

Antrocin was kindly provided by Dr. Yew-Min Tzeng (National Taitung University, Taiwan) as described previously [14]. Briefly, antrocin was isolated from the fruiting bodies of *A. cinnamomea* through a serial solvent extraction and silica gel column chromatography operations to yield antrocin as colorless crystals. The spectral data of ^1^H and ^13^C NMR derived from the antrocin were in complete accord with the assigned structure. The isolation method and all of the NMR spectral data of the natural product antrocin have been reported by Dr. Tzeng’s laboratory [14]. The purity (>95%) of the isolated antrocin was confirmed from its sharp melting point, TLC on silica gel (one spot), ^1^H and ^13^C NMR studies, and HPLC chromatographic analysis.

### 2.4. Ionizing Radiation

PCa cells and mice were irradiated in ambient air by using the Faxitron RX-650 irradiator (Faxitron X-ray, Wheeling, IL, USA) [17] at various doses (0, 2, 4, and 6 Gy), which are indicated in the experiments.

### 2.5. Cell Proliferation Assay

Cells (5 × 10^3^) were seeded in 96-well plates and treated with various concentrations of antrocin (0, 1, 5, 10, 20, 50, 100, 200, and 500 μM), followed by incubation for 24, 48, and 72 h. After the indicated times of incubation, 10 μL of 3-(4,5-dimethylthiazol-2-yl)-2,5-diphenyl tetrazolium bromide (MTT) were added to the cells and incubated for an additional 4 h at 37 °C. Acid-isopropanol (0.04 M HCl/isopropanol) was added to each well to effectively dissolve the formazan crystals [18]. The absorbance was measured by a microtiter plate reader (Model 680, BioRad, Hercules, CA, USA) at the wavelength of 562 nm.

### 2.6. WST-1 Cell Cytotoxicity Assay

At the indicated time, 10 μL of WST-1 (Roche Molecular Biochemicals, Basel, Switzerland) were added to each well at a ratio of 1:9 (*v*/*v*). The treated cells were incubated at 37 °C for 4 h, as recommended by the manufacturer. The plates were then placed on a shaker for 5 min and the absorbance was determined by a microplate reader at 420–480 nm.

### 2.7. Clonogenic Cell Survival Assay

The vehicle or IR-treated cells were plated in each petri dish and 1000 cells per dish were plated for the antrocin alone or antrocin plus IR treatments [19]. Thereafter, the cells were treated with the vehicle control, IR (2–6 Gy), and/or antrocin (100 μM). Cells were then incubated for seven days, the colonies were fixed with 4% formaldehyde and stained with 0.05% crystal violet in PBS. Cell survival was assessed by the standard colony formation assay as described previously [11]. The data are presented as the mean ± SEM of three independent experiments. The colony survival curve S = e^(αD+ßD2)^ was fitted to the experimental data using the least squares fitting algorithm. The data were analyzed and survival curves were plotted following the linear quadratic (LQ) model using GraphPad Prism 6.0 software (GraphPad Software, San Diego, CA, USA).

### 2.8. Wound-Healing Migration Assay

PC3-KD cells were seeded in 6-well plates at a density of approximately 3 × 10^5^ and were cultured to reach 100% confluence for 24 h. Two perpendicular scratches were performed by using a sterile p200 pipette tip. The media were then replaced and incubated for an additional 20 h. Serial images were obtained at 0, 5, 10, and 20 h. The image was measured for all fields of each well by using ImageJ (National Institute of Health, Bethesda, MD, USA) [20].

### 2.9. Cell Cycle Analysis

Cell cycle distribution was assayed by determining the DNA content. PC3-KD cells were treated with the vehicle control, IR (2 Gy), and/or antrocin (100 μM), followed by incubation at 37 °C for 48 h. Cells were then fixed with ice-cold 70% ethanol overnight and stained with 20 μg/mL propidium iodide (Sigma-Aldrich) containing 1 mg/mL RNase (Sigma-Aldrich) and 0.1% Triton X100 for 30 min. The stained cells were determined by an FACScalibur flow cytometer (BD FACSCalibur, BD Biosciences, Bedford, MA, USA) and the data were analyzed using Cell Quest software WinMDI (Verity Software House, Topsham, ME, USA). Apoptotic cells were counted and represented as a percentage of the total cell count as described previously [17].

### 2.10. Western Blot Assay

PC3-KD cells were seeded in 6-cm dishes and the assigned experiments were performed. Cells treated with antrocin, IR, or antrocin combined with IR for 48 h were harvested. Cells were lysed with 100 μL RIPA reagent with protease and phosphatase inhibitors (Roch, Indianapolis, IN, USA), and the cell lysates were prepared to perform the Western blot assay. The samples were then resolved by 10% or 12% SDS-PAGE and transferred onto a polyvinylidene difluoride (PVDF) membrane (Millipore, Billerica, MA, USA). After the PVDF membrane was incubated with 5% dehydrated skim milk to block nonspecific protein binding. The membrane was probed with primary antibodies as indicated in each experiment and then incubated with the HRP-conjugated secondary antibody. The proteins of interest were detected using ECL Western Blotting Detection Reagents (RE Healthcare, Little Chalfont, UK) and visualized using Image Quant LAS4000 and TL software (GE Healthcare; Chicago, IL, USA). The signal intensity of each protein was quantified with UN-SCAN-IT software (Silk Scientific Corporation, Orem, UT, USA).

### 2.11. Lentiviral Vector Production and Short Hairpin RNA (shRNA) Transfection

shRNA lentiviral particles and shRNA were purchased from National RNAi Core Facility (Academia Sinica, Taipei, Taiwan). The targeting sequence for ZNF443 (TRCN0000019855 and TRCN0000414601), TNFSF13B (TRCN0000058550 and TRCN0000358826), COL2A1 (TRCN0000083623 and TRCN0000083625), and TMX1 (TRCN0000147353 and TRCN0000338583) shRNA were CCCAGTTCACTTCAAACACAT, GTTCGAGCTCCTTTCGATATC, GTGACTTTGTTTCGATGTATT, GACAGTGAAACACCAACTATA, CCTGACCTGATGTCCA TTCAT, GCTGGCTTCAAAGGTGAACAA, GCTGAAAGTAAAGAAGGAACA, and GCTGAA AGTAAAGAAGGAACA, respectively. The sequence for the control shRNA (TRCN0000072249) was GCGGTTGCCAAGAGGTTCCAT. Lentivirus was packaged in HEK293T cells. PC3-KD cells were then infected with recombinant lentiviruses, and stable cell lines were selected with puromycin (0.4 μg/mL) for three days.

### 2.12. Analysis of Microarray Expression Profiling

PC3-KD cells were treated with the vehicle control, antrocin (100 µM) alone, IR (2 Gy) alone, or antrocin plus IR for 48 h. Total RNA were isolated using a TRIzol RNA isolation kit (Rockville, MD, USA) according to the manufacturer’s protocols. The array was analyzed by using the clusterProfiler software [21]. Differential gene expression analysis among the vehicle control, IR, antrocin, and antrocin plus IR-treated samples were subsequently conducted using the Bioinformatics & Evolutionary Genomics Analysis (http://bioinformatics.psb.ugent.be/webtools/Venn/) [22]. Data were normalized and selected by determining a *P*-value less than 0.05, and 2-fold changes (Table S1). Gene network and pathway analysis was performed by using GeneMANIA (http://www.genemania.org) [23]. The Heatmap analysis was using gplots: Various R Programming Tools (https://cran.r-project.org/web/packages/gplots/index.html) [24].

### 2.13. Isolation of RNA and Quantitative Real-Time Reverse Transcription PCR

Total RNA was isolated from cells using an RNeasy Mini Kit (QIAGEN, Valencia, CA, USA) and then reverse transcribed into cDNA using the oligo(dT) primer. Quantitative real-time PCR using a SYBR Green I Master Mix and a model 7900 Sequence Detector System was conducted to the manufacturer’s instructions (Applied Biosystems, Foster, CA, USA). The primer sequences were designed (Table S2) and subjected to qRT-PCR analysis. After pre-incubation at 60 °C for 2 min and 95 °C for 10 min, PCR was performed with 40 cycles of 95 °C for 15 s and 60 °C for 1 min. The threshold was set above the non-template control background and within the linear phase of target gene amplification to calculate the cycle number at which the transcript was detected (denoted as C*_T_*).

### 2.14. Animal Study

Male nude mice (BALB/cAnN.Cg-Foxnlnu/CrlNarl, National Laboratory Animal Center) at 3-weeks old were used in this study. The mouse care and treatment were performed according to the protocols reviewed and performed in accordance with the Animal Care and Use Guidelines for Chang Gung University. The approval number from Chang Gung University is CGU15-121, and the permission date is 29 December 2015. The mice were allowed to acclimate for an additional two weeks in the animal facility before any intervention was initiated. A suspension of LAPC4-KD (2 × 10^5^) tumor cells was placed in a 50% mixture of Matrigel (BD Biosciences, Bedford, MA, USA) in 0.1 mL was injected subcutaneously into the right posterior flanks of the mice. Mice were divided into four groups (five animals/group) including the vehicle control, IR alone (2 Gy), antrocin alone (100 mg/kg), and combined treatment of antrocin with IR. The dose of each administered irradiation was 2 Gy. The mice were treated with a total dose of 14 Gy delivered in seven fractions using the Faxitron RX-650 irradiator (Faxitron X-ray, Wheeling, IL, USA) on days 1, 3, 5, 7, 9, 11, and 13. Tumors were measured three times per week using Vernier calipers. The tumor volume was estimated by length × height × width × 0.5236 as described previously [25].

### 2.15. Immunohistochemistry (IHC) Analysis

Tissue specimens from mice were formalin-fixed and then subjected to IHC staining as described previously [25]. Briefly, the tissue sections were de-waxed and rehydrated. The sections were then stained with monoclonal antibodies against cyclin-D1, cleaved PARP, cleaved-caspase 9, phospho-AKT, and phospho-β-catenin (Cell Signaling) for 24 h at 4 °C. The samples were then probed with peroxidase-labeled goat anti-rabbit secondary antibody (Epitomics, Burlinggame, CA, USA) and detected with an ABC kit (Vector Laboratories, Burlingame, CA, USA).

### 2.16. Statistical Analysis

Statistical analyses for the data between two groups were determined using post-hoc *t*-tests. Statistics analysis comparisons of more than two groups were evaluated using two-way analysis of variance (ANOVA). The statistical analysis was performed by using the SPSS program (version 18.0 for windows, SPSS Inc., Chicago, IL, USA). *p* < 0.05 was considered statistically significant.

## 3. Results

### 3.1. Antrocin Induces Cell Death by Increasing Sensitivity to IR

**Figure 1:**
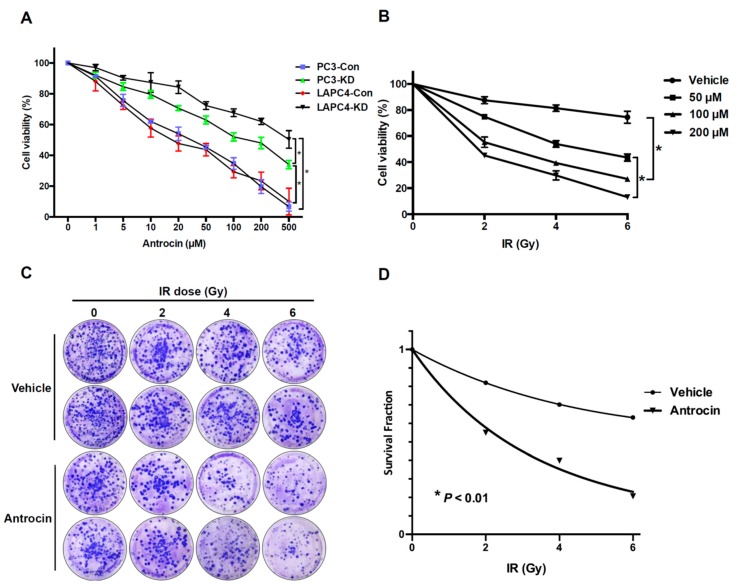
Antrocin inhibits PCa cells proliferation and sensitizes PCa cells to IR-induced cell death. (**A**) PC3-Con, PC3-KD, LAPC4-Con, and LAPC4-KD cells were treated with various concentrations of antrocin (0, 1, 5, 10, 20, 50, 100, 200, and 500 μM) for 48 h. An approximately half-maximal inhibition of cell viability was obtained at 100 μM antrocin concentration. (**B**) PC3-KD cells were exposed to IR (0–6 Gy) and antrocin (50, 100, or 200 μM, respectively) followed by incubation for 48 h. Cell proliferation was assessed by using the MTT assay. (**C**) PC3-KD cells were treated with IR (0–6 Gy) alone or co-treatment IR and antrocin (100 μM). After 7-day incubation, survival cell colonies were stained with crystal violet and (**D**) assessed through clonogenic assays as described in the Methods Section. Statistical significance was evaluated by two-way ANOVA (* *p* < 0.01).

DAB2IP-knockdown (KD) radioresistant cell lines (LAPC4-KD and PC3-KD) and shControl cell lines (LAPC4-Con and PC3-Con) were incubated with various concentrations of antrocin (0, 1, 5,10, 20, 50, 100, 200, and 500 μM) for 48 h. As shown in Figure 1A, antrocin effectively inhibited LAPC4-KD and PC3-KD cell proliferation in a dose-dependent manner. Our results also showed that antrocin markedly inhibited cell proliferation in a dose-dependent manner in several parental PCa cell lines including PC3, DU145, and LNCaP, while its effect on PZ-HPV-7 cells was negligible (Figure S1). As antrocin induced considerably higher cytotoxicity in PC3-KD cells than in LAPC4-KD cells, we chose to use PC3-KD cells for the rest of the study. We then investigated whether antrocin enhances radiosensitivity in PCa cells. PC3-KD cells were treated with antrocin (0–200 μM) followed by exposure to IR (0–6 Gy) before cell viability was determined. Our data showed that the cell viability was slightly decreased in the IR-treated group, while antrocin and IR co-treatment significantly reduced cell viability in a dose-dependent manner (Figure 1B). These results reveal that antrocin increased the effects of radiation in PC3-KD cells.

We then examined whether antrocin increases the susceptibility of PCa cells to radiation. PC3-KD cells were treated with IR (0–6 Gy) alone or combined with antrocin (100 μM) before we subsequently analyzed cell survival through a clonogenic assay. As shown in Figure 1C,D, antrocin synergistically enhanced the radiosensitivity of PC3-KD cells with increasing IR doses. Cells treated with the combination of antrocin and IR were remarkably more susceptible to IR-induced cell death than the cells treated with IR alone. These results demonstrate that antrocin effectively sensitized PCa cells to radiation.

### 3.2. Antrocin Suppresses Cell Migration and Arrests Cell Cycle in PCa Cells

**Figure 2:**
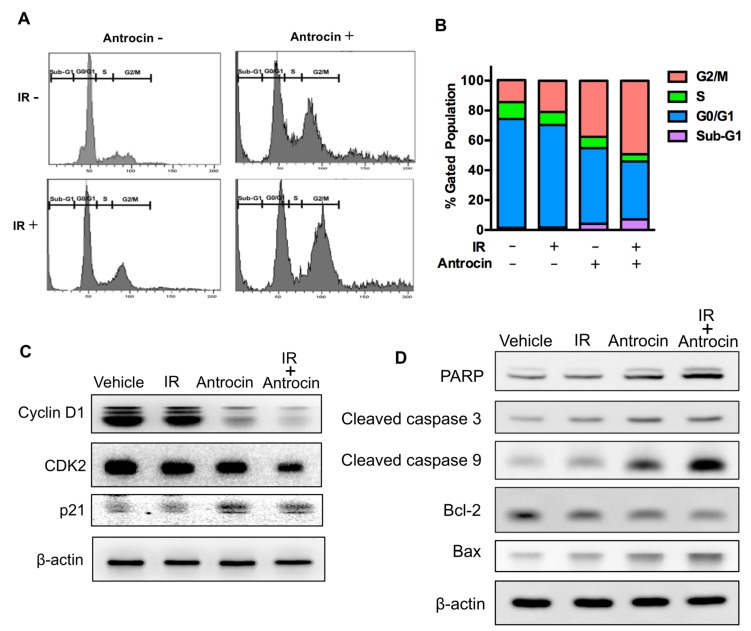
Antrocin and radiation synergistically arrest the cell cycle at G2/M and regulate apoptosis-related molecules in radioresistant PCa cells. (**A**) PC3-KD cells were treated with the vehicle control, IR (2 Gy) alone, antrocin (100 μM) alone, or IR combined with antrocin, and incubated for 48 h. Cell cycle distribution based on DNA content was analyzed through flow cytometry. (**B**) Different cell phases were plotted as the percentage of total events. (**C**,**D**) Cell lysates were harvested and subjected to Western blot analysis to determine the protein expression levels. The data represent one of three independent experiments. β-actin was used as a loading control.

To investigate the effect of antrocin on the cell migration activity of PCa cells, a wound-healing assay was performed. As shown in Figure 2, the migration activities of PCa cells were significantly repressed in antrocin-treated groups (100 and 200 μM) when compared to those in the control groups (*p* < 0.01). To further assess whether antrocin inhibits cell cycle progression, PC3-KD cells were treated with IR (2 Gy) alone or in combination with antrocin (100 μM) for 48 h, followed by cell cycle analysis. Our results showed that the treatment of cells with IR alone slightly induced G2/M arrest when compared to the vehicle control cells (Figure 2A,B). However, co-treatment with IR and antrocin markedly arrested the cell cycle at the G2/M phase and increased the sub-G1 population. These results indicate that antrocin synergistically enhanced IR-induced cell death in radioresistant PCa cells by inducing cell cycle arrest. 

### 3.3. Antrocin Inhibits Cell Cycle Regulatory Molecules and Induces Apoptosis in PC3-KD Cells

Since cell division is regulated by cyclins, cyclin-dependent kinases (CDKs), and CDK inhibitors such as p21 [26], we then investigated the effect of antrocin on these cell cycle regulators. PC-3 KD cells were treated with IR (2 Gy) alone, with antrocin (100 μM) alone, or with a combination of the two for 48 h; then, the expression levels of cyclin D1, CDK2, and p21 were analyzed through Western blot. As shown in Figure 2C, IR and antrocin reduced the levels of cyclin D1 and CDK2 but increased the levels of p21 in PC3-KD cells. We then characterized the key molecules that were involved in apoptosis. Markedly increased levels of activated PARP, cleaved-caspase 3, and cleaved-caspase 9 were observed in cells treated with antrocin plus IR (Figure 2D). In addition, antrocin and IR significantly inhibited the expression of the anti-apoptotic molecule, Bcl-2, but increased the expression of the apoptosis-promoting molecule, Bax (Figure 2D). These results demonstrate that antrocin is a potent agent for increasing IR-induced apoptosis in radioresistant PCa cells.

### 3.4. Microarray Profiling in Antrocin-Treated Radioresistant PCa Cells

**Figure 3:**
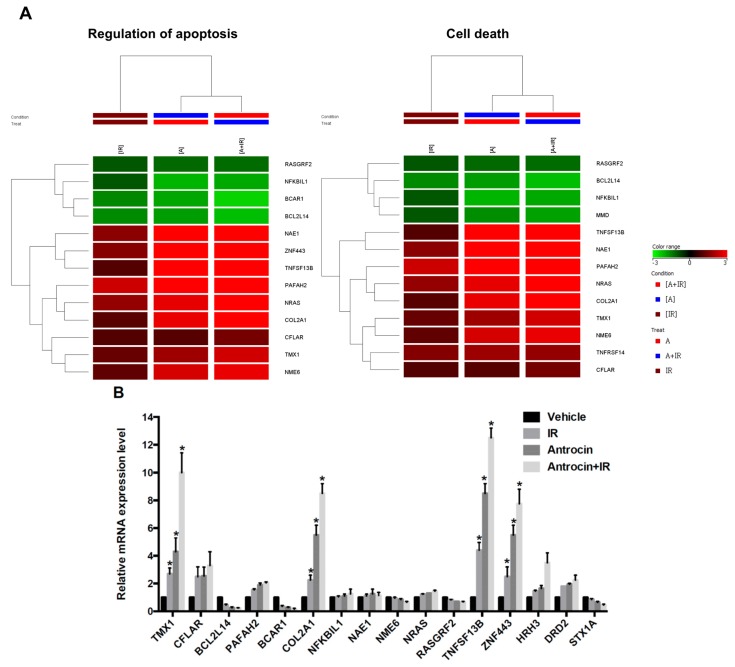
Gene expression profiles in PCa cells treated with IR and antrocin. After PC3-KD cells were treated with antrocin (100 μM) and/or IR (2 Gy), or co-treatment with IR and antrocin, a microarray analysis was performed. (**A**) Heatmap analysis showed the gene expression changes. Analysis of apoptosis-related genes are shown. (**B**) Apoptosis-related genes that were differentially expressed between treated and untreated groups were validated through qRT-PCR. Average fold-changes are presented. Statistical significance was evaluated by two-way ANOVA (* *p* < 0.01).

To further explore the mechanism of antrocin-mediated cell death, PC3-KD cells were treated with IR (2 Gy) alone, antrocin (100 μM) alone, or a combination of the two for 48 h. From the cells, mRNA was isolated and gene expression was subsequently analyzed using clusterProfiler [21]. Genes with a *P*-value less than 0.05 were considered differentially expressed relative to the vehicle-treated controls. Heatmap analysis showed that the expression levels of apoptosis and cell death-related pathways were consistently upregulated in cells treated with IR and antrocin when compared to those in the vehicle control cells (Figure 3A) [24]. Microarray analysis showed 16 genes were differentially expressed in four groups including *TMX1*, *CFLAR*, *BCL2L14*, *PAFAH2*, *BCAR1*, *COL2A1*, *NKBIL1*, *NAE1*, *NME6*, *NRAS*, *RASGRF2*, *TNFSF13B*, *ZNF443*, *HRH3*, *DRD2*, and *STX1A*. We then examined the mRNA expression levels through qRT-PCR and validated that *ZNF443*, *TNFSF13B*, *COL2A1*, and *TMX1* were the most highly upregulated genes in the cells treated with IR and antrocin (Figure 3B). These genes are involved in apoptosis or cell death regulation and were highly consistent across microarray and qRT-PCR experiments. Therefore, we explored the roles of these genes in the antrocin-induced sensitization of IR-induced cell death in radioresistant PCa cells.

### 3.5. Involvement of ZNF443, TNFSF13B, COL2A1, and TMX1 in Antrocin-Enhanced Radiosensitivity in PCa Cells

**Figure 4:**
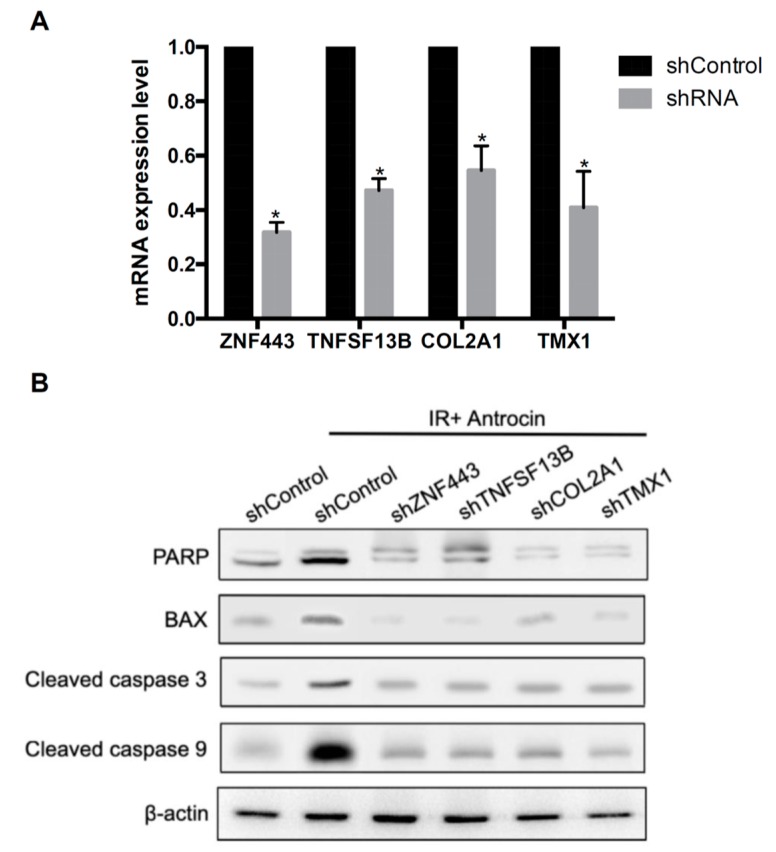
Knockdown of *ZNF443*, *TNFSF13B*, *COL2A1*, and *TMX1* decreases antrocin and IR-induced apoptosis-related molecule expression. (**A**) mRNA levels in shRNA-Control (shControl) and shRNA knockdown cells were analyzed using qRT-PCR. GAPDH was used as an internal control. * *p* < 0.01 when compared to each group. (**B**) PC3-KD cells were transfected with shControl or with gene-targeting shRNA. The shControl or shRNA knockdown cells were vehicle-treated or co-treated with IR (2 Gy) and antrocin (100 μM) for 48 h. Cell lysates were harvested and subjected to Western blot analysis. The data represent one of three independent experiments. β-actin was used as a loading control.

To investigate whether *ZNF443*, *TNFSF13B*, *COL2A1*, and *TMX1* are involved in the antrocin induction of the susceptibility of PCa cells to radiation, specific lentivirus-delivered shRNA against *ZNF443*, *TNFSF13B*, *COL2A1*, and *TMX1* genes were used to knockdown the respective genes. The mRNA levels of *ZNF443*, *TNFSF13B*, *COL2A1*, and *TMX1* in the transduced cells were analyzed by qRT-PCR. As shown in Figure 4A, *ZNF443*, *TNFSF13B*, *COL2A1*, and *TMX1* had markedly decreased mRNA expression levels in the PCa cells treated with the shRNAs when compared to the shControl cells. To further investigate the influence of the shRNA knockdown of genes on antrocin- and IR-induced cell death, Western blot analysis was performed to evaluate the expression levels of apoptotic molecules. As shown in Figure 4B, compared to the control shRNA (shControl) cells without treatment, the protein expression levels of PARP, Bax, cleaved-caspase 3, and cleaved-caspase 9 were drastically elevated upon co-treatment with IR and antrocin in the shControl cells. However, the expression levels of the apoptotic molecules were remarkably decreased in each specific shRNA-transduced line. These results demonstrate that knockdown of *ZNF443*, *TNFSF13B*, *COL2A1*, and *TMX1* suppressed antrocin/IR-induced apoptosis of PCa cells.

### 3.6. Antrocin Inhibits the Downstream Signaling of PI3K/AKT and MAPK Pathways

**Figure 5:**
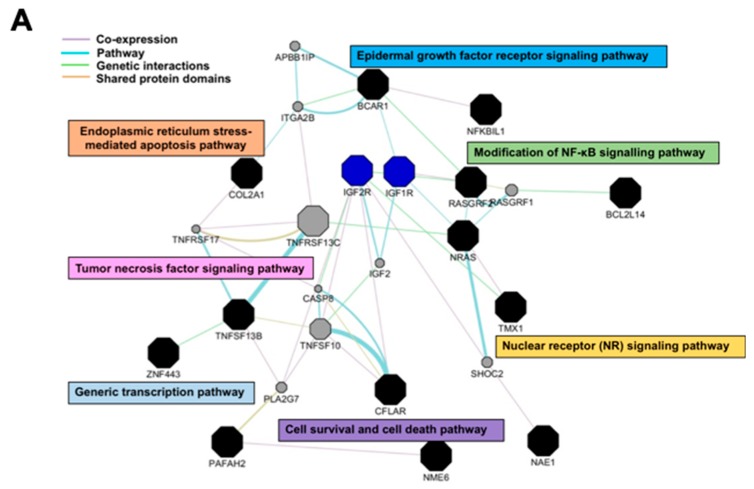

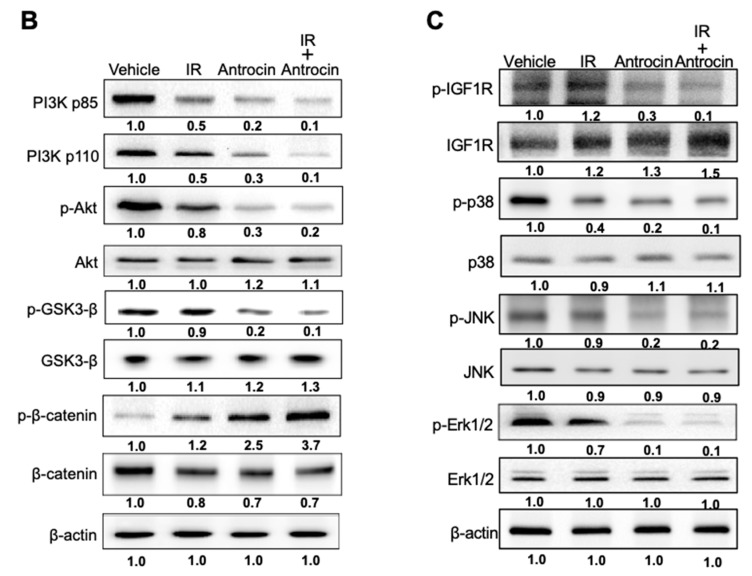
Antrocin inhibits the PI3K/AKT and MAPK signaling pathways and phosphorylation of IGF-1R. PC3-KD cells were treated with IR (2 Gy), antrocin (100 μM), or IR plus antrocin, followed by incubation for 48 h. (**A**) The represented gene network was identified by ingenuity pathway analysis. The color of each line indicates the regulation of gene expression. (**B**) Cell lysates were harvested and subjected to Western blot analysis using antibodies against p85, p110, p-AKT, AKT, GSK3-β, p-GSK3-β, p-β-catenin, and β-catenin, and (**C**) antibodies against IGF-1R, p38, JNK, Erk1/2, and their respective phosphorylated forms. The data represent one of three independent experiments. β-actin was used as a loading control. The expression level of each protein was quantified by the signal intensity and normalized with each vehicle untreated group. The relative level of each protein expression was indicated at the bottom of each lane.

The top-scoring pathways containing differentially regulated genes were analyzed using GeneMANIA to visualize the interaction network for the genes in these categories [23]. Pathway analysis showed marked enrichment for genes that were associated with cell proliferation and apoptosis pathways (Figure 5A). The results from the interaction network analysis indicated that IR and antrocin treatments were associated with IGF-1R, which is involved in the regulation of several major phosphorylation cascades including the PI3K and MAPK pathways [27,28]. Among the known apoptotic pathways, the PI3K signaling pathway plays an important role in cell survival and proliferation [29]. In addition, the PI3K signaling is one of the most commonly deregulated pathways in PCa development [30]. We therefore determined the expression levels of IGF-1R and other molecules that were involved in the PI3K/MAPK pathway. Our results showed a significant decrease in the expression of phospho-IGF-1R, phospho-p38, phospho-JNK, and phospho-Erk1/2 in cells treated with IR and antrocin when compared to that in the vehicle control cells. We then showed that co-treatment with IR and antrocin reduced the expression of PI3K, phospho-AKT, and phospho-GSK3-β, but elevated phospho-β-catenin expression (Figure 5B,C). It is known that AKT-mediated phosphorylation of the β-catenin signaling pathway is involved in the induction of apoptosis [31]. Together, these results indicate that antrocin enhanced IR-induced apoptosis by inhibiting the downstream signaling of the PI3K/AKT and MAPK pathways.

### 3.7. Antrocin Synergistically Enhances IR-Suppressed PCa Growth In Vivo

**Figure 6:**
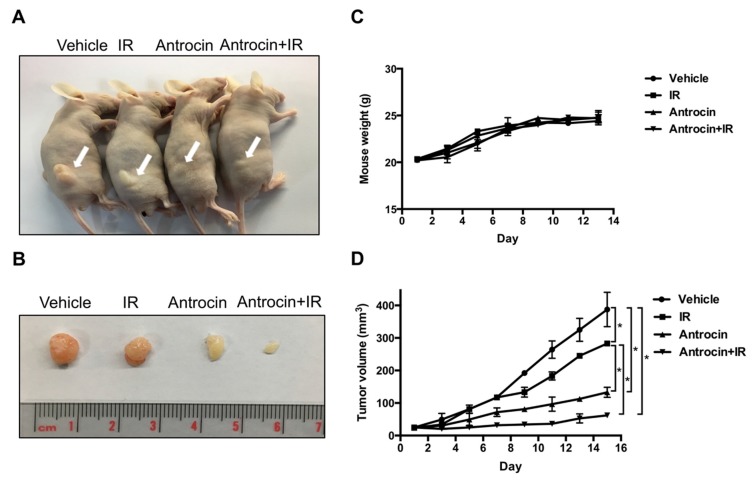
Antrocin synergistically enhances radiation-suppressed PCa growth in vivo. (**A**) Mice with xenograft tumors were divided into four groups: vehicle-treated, treated with IR (2 Gy), treated with antrocin (100 mg/kg), or treated with a combination of IR and antrocin, respectively. Arrows indicate tumors that were grown in the posterior flanks of mice. (**B**) Treatments were administered on days 1, 3, 5, 7, 9, 11, and 13. After euthanasia, tumors were excised from the mice. Scales shown in images are in centimeters. (**C**) Mouse weights were recorded. (**D**) Tumor volume was measured, and data are presented as means ± SEM. Vehicle vs. IR: *p* = 0.0264; Vehicle vs. Antrocin: *p* = 0.0177; Vehicle vs. Antrocin+IR: *p* = 0.0116; IR vs. Antrocin: *p* = 0.0211; IR vs. Antrocin+IR: *p* = 0.0192. Statistical significance was evaluated by two-way ANOVA (* *p* < 0.01).

**Figure 7:**
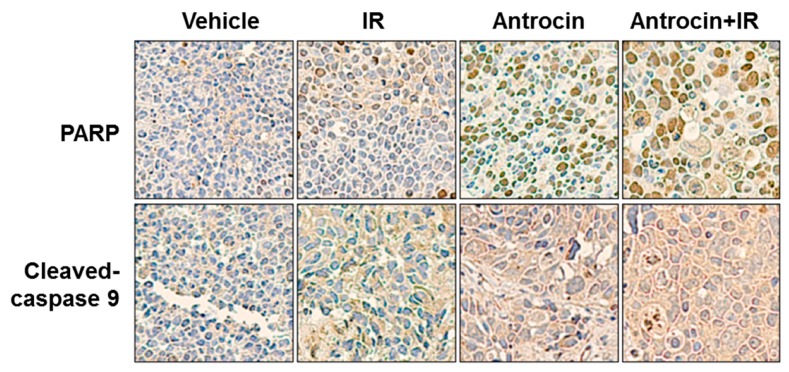

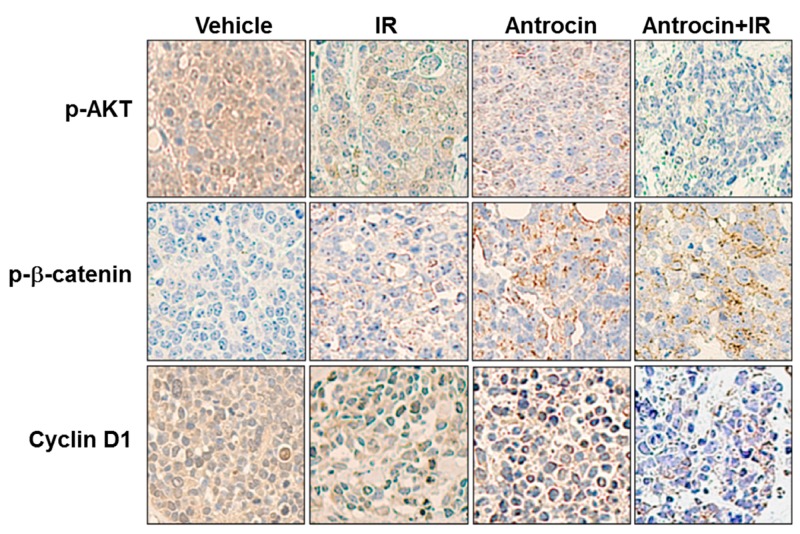
Representative immunohistochemical (IHC) staining patterns in xenograft PCa tissue. IHC analysis of paraffin sections show staining with specific antibodies against PARP, cleaved-caspase 9, phospho-AKT, phospho-β-catenin, and cyclin D1, respectively. Images were photographed at 200× magnification.

**Figure 8:**
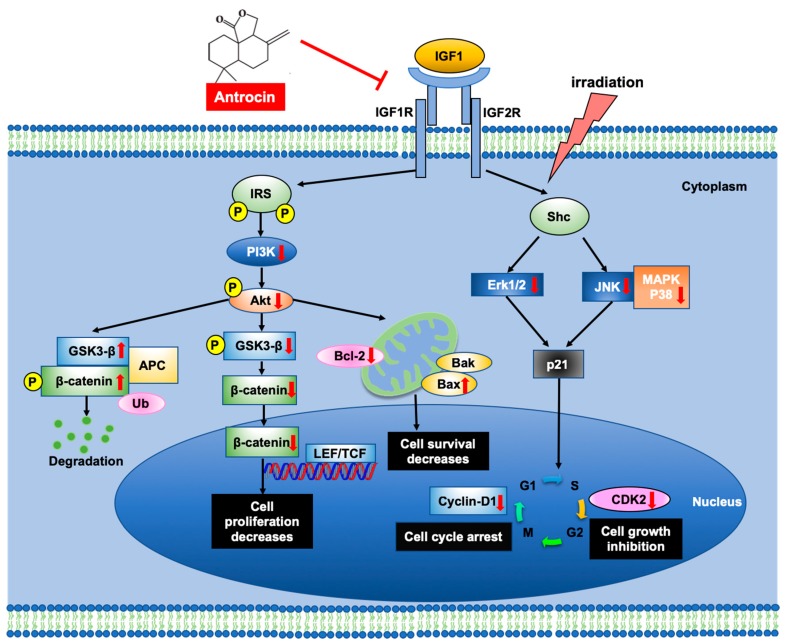
Proposed mechanism of action for antrocin-induced sensitization to radiotherapy in radioresistant PCa cells. IGF-1 binds to IGF-1R and induces IRS/Shc phosphorylation, which leads to the activation of PI3K/AKT and MAPK signaling. Co-treatment of cells with antrocin and radiation inhibits IGF1-induced activation of the PI3K/AKT and MAPK pathways, effectively sensitizing PCa cells to radiotherapy. IRS: insulin receptor substrate; Shc: src homology/collagen; LEF: lymphoid enhancer factor; TCF: transcription factor; Ub: ubiquitin.

To further evaluate the efficiency of antrocin in sensitizing PCa cells to radiation, we established PCa xenograft mouse models. The tumors were treated with the vehicle control, IR (2 Gy), antrocin (100 mg/kg), or a combination of IR and antrocin. As shown in Figure 6, mice treated with IR alone showed tumor growth that was slightly reduced when compared to that of the vehicle-treated control. However, treatment of IR and antrocin dramatically inhibited tumor growth. Compared to the vehicle-treated controls, mice treated with antrocin alone and antrocin plus IR resulted in a 76.4% and 88.2% reduction of tumor volume, respectively. We then analyzed the expression of cell cycle and apoptotic markers in the tumor tissues by immunohistochemistry and Western blot analysis. Our data showed that the expression levels of cyclin D and phospho-AKT were decreased in mice treated with a combination of IR and antrocin when compared to the vehicle controls. In contrast, the expression of cleaved-PARP, cleaved-caspase 9, and phospho-β-catenin increased in tumors co-treated with IR and antrocin when compared to that in the vehicle-treated control mice (Figure 7). We then analyzed the protein expression in tumor tissues by using Western blot assay. Our results showed that the expression levels of phospho-AKT and cyclin D were downregulated; however, cleaved-caspase 9 was upregulated in mice co-treated with IR plus antrocin in comparison with the vehicle-treated control mice (Figure S3). These results from cell-based and in vivo studies demonstrate that antrocin sensitizes radiation-induced cell death, which is mediated by an apoptotic mechanism associated with the inhibition of the PI3K/AKT and MAPK signaling pathways (Figure 8).

## 4. Discussion

*A. cinnamomea* is a fungal parasite on the inner cavity of the endemic species *Cinnamomum kanehirae* in Taiwan [32], and has been demonstrated to possess potent anti-proliferative activity against various cancers in vitro and in vivo [12]. Approximately 78 compounds from *A. cinnamomea* have been identified and structurally elucidated [12]. Chemical compounds found in *A. cinnamomea* include sesquiterpene lactones, steroids, and triterpenoids [33]. Antrocin can be isolated from *A. cinnamomea* with methanol under reflux and reduced pressure [14].

Antrocin is a potent antagonist against various cancer types including lung, breast, and bladder cancers [14,15,16]. Antrocin has been demonstrated to be a small-molecule inhibitor of AKT/mTOR/GSK-3β/NF-κB signaling in metastatic breast cancer cells [14]. In addition, antrocin can downregulate the JAK/STAT pathway and increase caspase-3 expression in non-small lung cancer cells [15]. In bladder cancer, antrocin inhibits migration and invasion by suppressing the ERK/FAK/paxillin and ERK/c-Fos/MMP-2 signaling pathways [16]. In this study, we further demonstrated that antrocin possesses potent activity to enhance radiation-induced apoptosis in radioresistant PCa cells. Inhibition of cell cycle regulatory proteins and accumulation of a G2/M population were observed in cells co-treated with antrocin and radiation. Furthermore, antrocin effectively inhibited tumorigenesis in PCa xenograft mice in vivo. Our results conclude that antrocin enhances radiation-induced apoptosis of PCa cells by the downregulation of PI3K/AKT and MAPK signaling pathways.

Microarray analysis showed that 16 genes were differentially expressed in the treatment groups. We then examined mRNA expression levels of the 16 genes by using qRT-PCR and found *ZNF443*, *TNFSF13B*, *COL2A1*, and *TMX1* as highly upregulated genes in cells treated with radiation plus antrocin when compared with the untreated control. The expression levels of these genes were significantly increased and remained consistent throughout the microarray and qRT-PCR experiments. Through silencing the mRNA of *ZNF443*, *TNFSF13B*, *COL2A1*, and *TMX1*, this decreased the antrocin and radiation-induced apoptosis-related molecules like PARP, Bax, cleaved-caspase 3 and cleaved-caspase 9. Among the genes we reported, *ZNF443* is known to enhance apoptosis following exposure to IR in hematopoietic cells [34]. *TNFSF13B* is involved in cell proliferation, differentiation, and apoptosis [35]. In addition, *TMX1,* as a thiol-based tumor suppressor, can increase mitochondrial ATP production and apoptosis progression [36]. Our results are in line with these findings, indicating that these genes are crucial for triggering apoptosis or cell death regulation in response to antrocin-enhanced radiosensitivity. 

sDNA-PKCs are associated with ATM, which play an important role in DSB repair signaling [37] A recent study reported that DAB2IP deficiency induced radioresistance due to the ATM-mediated enhancement of DSB repair kinetics, and the activation of the NF-κB and MAPK signaling cascades following radiation [38]. Furthermore, cyclin D1 regulates phosphorylation/activation of radiation-induced ATM and DNA-PKCs, which are associated with DSB repair in PCa [39]. Although we did not perform DNA-PKCs-related experiments, the expression of cyclin D1 and MAPK signaling was assessed. Our results indicate that antrocin sensitizes radiation therapy in PCa by arresting cell cycle and activating the apoptotic pathway. 

Glycogen synthase kinase-3β (GSK-3β), a target of phospho-AKT, regulates the stability of β-catenin [40]. The phosphorylation of GSK-3β is regulated by the activation of PI3K and AKT [40]. Inhibition of phospho-GSK-3β increased the proteosomal degradation of β-catenin and decreased the nuclear accumulation of β-catenin [41]. β-catenin plays an important role in cellular signaling by promoting the activation of transcription factors, T-cell factor (TCF), and lymphoid-enhancer factor (LEF), which trigger genes in the regulation of cell cycle (e.g., cyclin D1) [42]. Our results showed that cotreatment of cells with radiation and antrocin effectively reduced the expression of PI3K/AKT and its downstream effector phospho-GSK-3β, which, in turn, is involved in the induction of apoptosis and cell cycle arrest. These findings unravel that antrocin-mediated PI3K/AKT inhibition with decreased phospho-GSK-3β activity corresponds to increased radiosensitization.

IGF-1 is regulated by IGF-1R/Src/MAPK/ERK signaling [43]. The mitogenic, anti-apoptotic, and other effects of IGF-1 are mediated by its interaction with IGF-1R, which is mainly expressed in the mammary epithelium [44]. Ligand binding and subsequent phosphorylation of IGF-1R triggers the activation of two major signaling cascades via the insulin receptor substrate 1 (IRS-1): the PI3K/AKT and RAF/MAPK pathways, which stimulate proliferation and protection from apoptosis [45]. It is important to note that nuclear translocation of IGF-1R is associated with resistance to target therapy and contributes to poor prognosis [46]. In contrast, inhibition of IGF-1R expression with the monoclonal antibody against IGF-1R, ganitumab, induces cytotoxicity of cancer cells [46]. In the present study, we showed a decrease of phospho-IGF-1R expression in PCa cells co-treated with radiation and antrocin. In line with previous reports, our results reveal that antrocin reduces IGF-1R expression, which may be a crucial factor for conferring radiosensitivity in PCa cells.

## 5. Conclusions

This study demonstrated that antrocin enhanced the radiosensitivity of PCa most likely by arresting cell cycle at G2/M and activating the apoptotic pathway. We have provided the first evidence that antrocin synergistically increases IR-induced cell death of radioresistant PCa cells by suppressing the downstream signaling of IGF-1R to inhibit the PI3K/AKT and MAPK pathways. These observations may provide insights into the mechanisms by which antrocin sensitizes PCa to radiotherapy as well as suggesting the possibility of antrocin as a potent therapeutic agent for overcoming radioresistant PCa.

## Supplementary Materials

The following are available online at http://www.mdpi.com/2072-6694/11/1/34/s1. Figure S1: Antrocin inhibits the cell proliferation of parental PCa cells, Figure S2: Antrocin inhibits the migration of PC3-KD cells, Figure S3: Co-treatment with IR and antrocin inhibits the expression of cell cycle and apoptotic markers in xenograft PCa tissue, Table S1: Microarray profiling and data analysis, Table S2: Primers that were used in qRT-PCR analysis.
