# Supplementary material for: Antrocin Sensitizes Prostate Cancer Cells to Radiotherapy through Inhibiting PI3K/AKT and MAPK Signaling Pathways
Source: Cancers (Basel). 2018 Dec 31;11(1):34. doi: 10.3390/cancers11010034 (PMC6356781; doi:10.3390/cancers11010034)
Supplement: Supplementary file 1 [file cancers-11-00034-s001.pdf]

Supplementary Materials: Antrocin Sensitizes Prostate Cancer Cells to Radiotherapy through Inhibiting PI3K/AKT and MAPK Signaling Pathways

Yu-An Chen, David T.W. Tzeng, Yi-Ping Huang, Chun-Jung Lin, U-Ging Lo, Chia-Lin Wu, Ho Lin, Jer-Tsong Hsieh, Chih-Hsin Tang and Chih-Ho Lai

Supplementary Materials

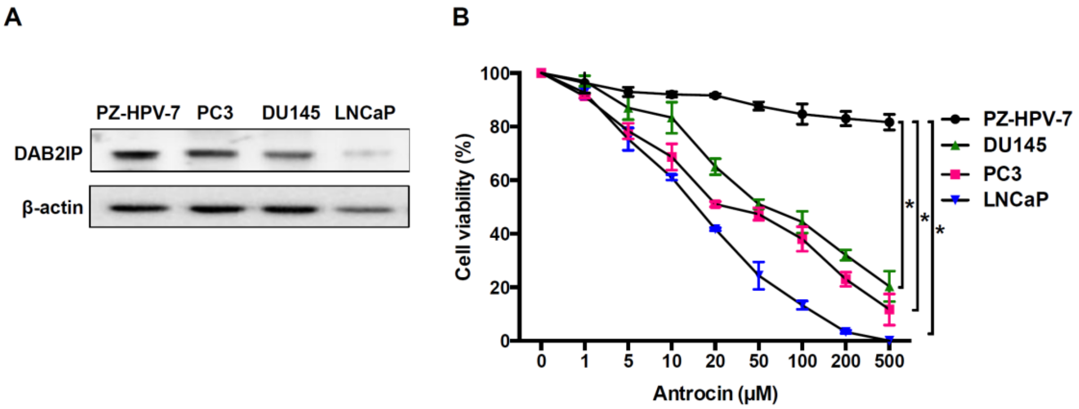

Figure S1. Antrocin inhibits the cell proliferation of parental PCa cells. (**A**) Cell lysates of PZ-HPV-7, PC3, DU145, and LNCaP were prepared for the analysis of DAB2IP expression using the Western blot assay. β-actin was used as a loading control. (**B**) PZ-HPV-7, PC3, DU145, and LNCaP cells were treated with various concentrations of antrocin (0, 1, 5, 10, 20, 50, 100, 200, and 500 μM) for 48 h. Cell proliferation was assessed by using the WST-1 assay. Statistical significance was evaluated by two-way ANOVA (*, $p < 0.01$).

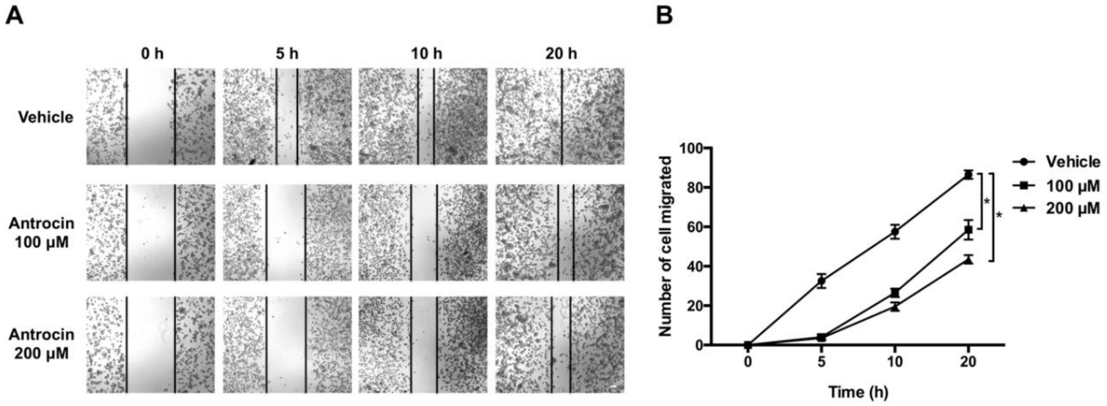

Figure S2. Antrocin inhibits the migration of PC3-KD cells. The migration activity of PCa cells was evaluated under a microscope (10× magnification) through a wound-healing assay. (**A**) The representative images of the wound-healing assay are displayed. After being scratched with a 200-μL pipette tip, PC3-KD cells were vehicle-treated or treated with antrocin (100 and 200 μM), then images were taken at 0, 5, 10, and 20 h. (**B**) Quantitative analysis of the migration inhibition assay. Statistical significance was evaluated by two-way ANOVA (*, $p < 0.01$). Scale bar, 100 μm.

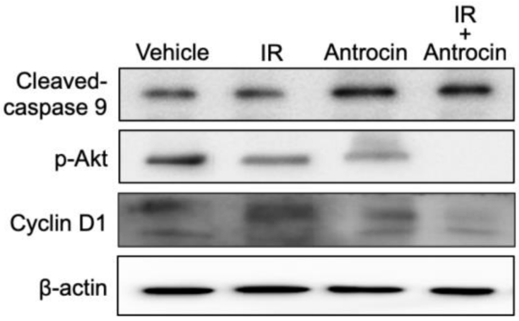

Figure S3. Co-treatment with IR and antrocin inhibits the expression of cell cycle and apoptotic markers in xenograft PCa tissue. The expression levels of cleaved-caspase 9, p-Akt, and cyclin D1 from tumor tissues were analyzed by Western blot assay. β-actin was used as a loading control.

Table S1. Microarray profiling and data analysis.

2x Up or Down	Fold change	Fold change	Fold change	Gene Symbol	Ensembl ID	Entrez GeneID	Genbank Accession	Genomic Coordinates
**Probe Name**	**C+IR vs Control**	**C+A vs Control**	**A+IR vs Control**					
A_23_P67278	3.08	24.96	31.99	ZNF443	ENST00000301547	10224	NM_005815	chr19:12541400-12541341
A_23_P14174	2.07	26.82	23.15	TNFSF13B	ENST00000486502	10673	NM_006573	chr13:108922693-108922752
A_33_P3283971	–2.09	–4.56	–4.09	NFKBIL1	ENST00000376148	4795	NM_005007	chr6:31526381-31526440
A_33_P3369034	–3.13	–3.99	–5.89	BCAR1	ENST00000393422	9564	NM_001170714	chr16:75263017-75262958
A_33_P3733417	2.12	5.53	5.35	DRD2	ENST00000362072	1813	NM_000795	chr11:113280398-113280339
A_23_P133386	–2.07	–2.37	–2.47	RASGRF2	ENST00000265080	5924	NM_006909	chr5:80521773-80521832
A_33_P3317825	3.50	6.90	13.34	NRAS	ENST00000369535	4893	NM_002524	chr1:115251224-115251165
A_23_P168556	2.29	2.00	2.10	STX1A	ENST00000222812	6804	NM_004603	chr7:73113602-73113543
A_23_P76951	2.41	3.69	5.72	TMX1	ENST00000457354	81542	NM_030755	chr14:51722559-51722618
A_23_P137765	5.48	10.09	16.32	PAFAH2	ENST00000374284	5051	NM_000437	chr1:26288337-26288278
A_23_P412990	–2.47	–2.60	–3.23	HRH3	ENST00000340177	11255	NM_007232	chr20:60790021-60790017
A_23_P500464	2.10	7.10	11.41	COL2A1	ENST00000380518	1280	NM_001844	chr12:48367008-48366949
A_23_P313961	2.27	5.89	7.12	NME6	ENST00000418431	10201	NM_005793	chr3:48336122-48336063
A_23_P116653	–3.18	–3.68	–4.95	BCL2L14	ENST00000396369	79370	NM_138722	chr12:12240295-12243746
A_23_P156807	2.01	2.07	2.70	CFLAR	ENST00000594734	8837	NM_001202519	chr2:202030660-202030717
A_23_P77459	3.28	44.81	55.78	NAE1	ENST00000563221	8883	NM_001018159	chr16:66836909-66836850

Table S2. Primers that were used in the qRT-PCR analysis.

Gene Name	Primers
TMX1	5′-CAATGAATCAACTGACCATTACG-3′<br>5′-GGAGAGGGCTTTAAAGAAAACTTG-3′
CFLAR	5′-CAGCATCCTAAGGCAGAAGAA-3′<br>5′-GGGAAAGGTGGGGAAAAGAT-3′
BCL2L14	5′-AGGGTCTCTCCTTCCAGCTC-3′<br>5′-TCTTTCCAACTGATCTCCTGAA-3′
PAFAH2	5′-TGCTGCTTGGATAACTGGGTA -3′<br>5′-TCCATCAAGGTCCCAGAAAG-3′
BCAR1	5′-CCAGTGCAGTTTGGTGTGTC-3′<br>5′-TTTTGCTCTTCCTTTTAAAAACTT-3′
COL2A1	5′-TTCGGACTTTTCTCCCCTCT-3′<br>5′-ACTCAGGGGGCATTTGACT- 3′
NFKBIL1	5′-AGATTGAGACCTGGGAGCTGAND-3′<br>5′-ATCCTTTCCCTTACCCTTCCTT-3′)
NAE1	5′-CTGCTCAAGAGGTCATCAAAAT-3′<br>5′-CAGACTAAAGCACAACCCGAAG-3′
NME6	5′-AGGGAGGTGTCCACTATGTAGC-3′<br>5′-AGCAGAAATGGCACTGTAAGC-3′
NRAS	5′-GACCAGACAGGGTGTTGAAGAT-3′<br>5′-TGAAAGTGGCTCTTTTCTGACA-3′
RASGRF2	5′-GTTTGGGTCAAAGACAGATGCT-3′<br>5′-CCCATCTGACATTCCCTTATGT-3′
TNFSF13B	5′-GGGACTGAAAATCTTTGAACCA-3′<br>5′-CCTTTTTGTATAGTTGGTGTTTCACT-3′
ZNF443	5′-CTCGACATAAAAGGACTCACACA -3′<br>5′-ATTCATACGGCTTCTCTCCA-3′
HRH3	5′-CTTTTCCATTCTGGGTGTTTTC-3′<br>5′-CCTCAGCAATTTTGTCTCTCTTG-3′
DRD2	5′-CTAAGAGGCTGCTGAAAACCAT-3′<br>5′-TGGAAGGTGACTCGTCAAAGTT-3′
STX1A	5′-CTCCCTCGGCTTTACTCCTG-3′<br>5′-TTTTCCTGGTACAGATACAAATGTT-3′
GAPDH	5′-AACGGATTTGGTCGTATTGGG-3′<br>5′- CAGGGGTGCTAAGCAGTTGG-3′

© 2018 by the authors. Submitted for possible open access publication under the terms and conditions of the Creative Commons Attribution (CC BY) license (http://creativecommons.org/licenses/by/4.0/).
